# Perceptions of Third-year Medical Students of a Non-resident Hospitalist Rotation

**DOI:** 10.7759/cureus.4214

**Published:** 2019-03-09

**Authors:** Sanjay Bhandari, Pinky Jha, Abhishek Thakur, Brian T Gooley, Joel J Lange, Hari Paudel, Michael Frank

**Affiliations:** 1 Internal Medicine, Medical College of Wisconsin, Milwaukee, USA; 2 Internal Medicine, Brookfield Heart and Vascular Clinic and Medical College of Wisconsin, Brookfield, USA; 3 Internal Medicine, Frank H. Netter MD School of Medicine, North Haven, USA; 4 Emergency Medicine, Medical College of Wisconsin, Milwaukee, USA

**Keywords:** non-resident hospitalist team, survey, medical students, perception

## Abstract

Introduction

Although medical students in the United States frequently rotate on a non-resident hospitalist team, there is a paucity of literature on their perceptions regarding such rotation. We sought to assess the perceptions of third-year medical students (M3s) regarding a non-resident hospitalist rotation.

Methods

We conducted a web-based survey of M3s who had previously rotated on a non-resident hospitalist service. We assessed their perceptions regarding patient assignments and complexities, rounding preferences, barriers to learning, and the benefits of the rotation. A descriptive analysis was performed on the responses.

Results

Out of 49 respondents, 47% preferred carrying a maximum of three patients during rounds and 57% preferred patients with fewer comorbidities. Fifty-one percent preferred rounding on all patients covered by the team as opposed to rounding on their assigned patients only. Despite several perceived benefits of the rotation, students also identified various barriers to learning while rotating in a non-resident hospitalist rotation.

Conclusions

Our study evaluated the perceptions of M3s regarding the ideal patient load, patient complexities, barriers to learning and various benefits of a non-resident hospitalist rotation. The students’ perceptions can potentially be addressed and incorporated to make the non-resident hospitalist rotation more educational for the students.

## Introduction

Hospitalists are board-certified internal medicine physicians who exclusively manage hospitalized patients. Since its inception more than two decades ago, the hospitalist field has grown exponentially, with more than 50,000 hospitalists practicing as of 2016 [1-2]. The success of the hospitalist model has received such widespread attention that the model has been adopted by 33 other countries in addition to the United States (US) and Canada [3]. Hospitalists have increasingly transformed the inpatient care patients receive through around-the-clock availability, reduced costs, increased patient satisfaction, and leadership roles in the areas of quality improvement and patient safety [4]. They also staff non-teaching services without residents, thereby offloading the physician crunch generated by the limitation on resident physician work hours [2]. Recent studies reveal similar or better patient outcomes with hospitalist-only services as compared to traditional teaching services [5-6]. A recent study done in an academic children’s hospital found no statistically significant differences in the outcomes of patients with common illnesses admitted to a hospitalist-only versus a teaching hospitalist service [5]. Another study performed in a university hospital revealed a better overall patient rating of a non-teaching hospitalist service as compared to a general medicine teaching service [6]. In addition to all the clinical responsibilities, hospitalists have also emerged as fundamental assets as clinician-educators in academic settings. Both residents and medical students perceive hospitalists as more favorable than non-hospitalists in terms of teaching effectiveness and overall satisfaction, as demonstrated by a systematic review [7].

During their internal medicine clerkship, medical students may rotate with the resident-covered "teaching" teams as well as resident-uncovered "non-teaching" hospitalist teams, which will be referred to as "non-resident hospitalist teams" in our study. An annual survey of clerkship directors in internal medicine showed that 91% of internal medicine clerkships use hospitalists as teaching physicians [8]. With recent restrictions on resident work hours, the non-resident hospitalist services are increasingly playing a vital role in providing direct supervision and teaching to the medical students during their internal medicine rotations. When assigned to a non-resident hospitalist service during an internal medicine clerkship, a student is typically paired one-to-one with a hospitalist physician. Students typically are given the responsibility to take histories from and perform physical examinations on a number of patients assigned to them and to take an active role in patient management while receiving direction and oversight from the hospitalist. While the majority of published studies so far have focused on trainee education on a traditional medicine team that includes both residents and medical students [9-11], there is a paucity of literature on the medical student experience on a non-resident hospitalist team.

Since third year is the typical time in medical school when medical students (M3s) get their first clinical exposure, a good clinical rotation during this period is of paramount importance in streamlining the students’ expectations for future medicine rotations, honing their first impression towards clinical medicine and helping cultivate their overall interest towards internal medicine, as well as playing a crucial role in their clinical education. In this study, we surveyed M3s who have previously rotated in a non-resident hospitalist service about their experience regarding the rotation. Participants were asked to provide their perceptions regarding the number and types of patients they think should ideally be assigned to them, as well as their perceived benefits, both clinical and educational, and different barriers to learning they encountered during this rotation. The study provides a template for future studies in this area examining the role of hospitalist-only services in medical education.

## Materials and methods

Study design, setting, and participants

We conducted an online survey of M3s at the Medical College of Wisconsin (MCW), a tertiary care academic medical center in the United States. Only the students who had completed their internal medicine non-resident hospitalist rotation between July 2017 and February 2018 participated in the survey.

Data collection and survey elements

The survey was conducted using the Qualtrics online-based survey platform (www.qualtrics.com). The survey was sent through an invitation email to all M3s along with the survey link. Only the students who had rotated on a non-resident hospitalist team were asked to participate. In addition to the actual survey questions, a free-written comment section was also allowed in case the students felt inclined to provide information that might not have been incorporated into the questions themselves. Survey questions included the general perceptions of the students regarding the hospitalist rotation, namely, the maximum number of patients that should be ideally assigned to them, the complexities of the patients to be accounted for, rounding preferences, opportunities to get involved in scholarly activities, their perceived barriers to learning, and the benefits of the rotation.

Data analysis

Descriptive statistics were used to summarize the responses with the use of respective frequencies and percentages. Analyses were conducted using SAS version 9.4 (SAS Institute, Cary, NC, US).

The study was approved by the institutional board review (IRB) at the Medical College of Wisconsin. The survey was voluntary, and the IRB approved the informed consent process through an informational letter sent to the participants through an invitation email, explaining the nature and expectations of the study and potential risks to the participants, along with a link to the survey. All possible steps were taken by the research team to maintain the anonymity of the participants.

## Results

A total of 49 students participated in the survey. With the total of 56 M3s who had rotated in the non-resident hospitalist teams over a period of eight months (from July 2017 through February 2018), this corresponded to a response rate of 87.5%.

**Figure 1:**
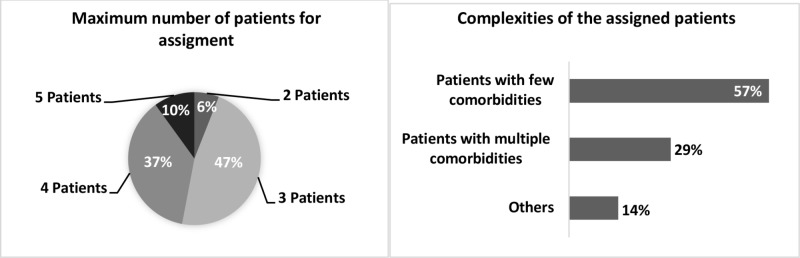
Perceptions of third-year medical students regarding the ideal patient load and complexity for assignment in a non-resident hospitalist rotation. ‘Others’ corresponds to the responses provided in the comment section.

A plurality of the students (23 out of 49 or 47% of the respondents) preferred three patients, at the maximum, to be assigned to them at a time (Figure 1).

Eighteen students (37%) thought four patients, at the maximum, would be ideal. While 10% said five patients would be an ideal number, only 6% said two patients would be preferable. None of the students responded to whether one patient would be an ideal number. The mean and mode of the maximum number of patients to be assigned to the students were 3.5 and 3, respectively.

Twenty-eight students (57%) thought that patients with fewer comorbidities were ideal for teaching and learning purposes while 14 (29%) preferred patients with multiple comorbid conditions (Figure 1). Six of the seven comments for this question suggested a mix of complex and simple patients (Table 1).

**Table 1:** Written comments provided by the third-year medical students regarding their perceptions of a non-resident hospitalist team.

Comments provided by the third-year medical students (unedited)
Complexities (simple versus complex patients):
1) A mix of both answers. Some bread and butter with some complex patients where there are several Consults on board as well.
2) Definitely some of each.
3) Both.
4) Mix of both. 1 hard patient and a few easy.
5) Patients presenting with symptoms that allow the student to do the work up themselves and figure out how to get to a diagnosis and treatment plan themselves.
6) Either one. There are things to learn from either case.
7) Mix of both.
Rounding Preferences (rounding on assigned patients only versus all patients in the team):
1) A mixture of rounding on assigned patients and other patients on the team who have significant teaching points.
2) Round on all patients with learning benefit even if you are not directly following them. But hospitalist round later on difficult disposition or placement patients.
3) What I have been doing is the mix of two. First I do "pre-round" on my own, and follow hospitalist's rounding later. He can start earlier if he prefers, but we round together the majority of patients after my "pre-rounds". It also gives me some independence, also I get to learn from the hospitalist.
4) Rounding on patients who may have clear physical exam findings or components to their case that the hospitalist will think is good for the student to learn.
Barriers:
1) No resident to ask logistical questions (how to call Consults, write discharge summary, make dotphrases on epic etc...) also I felt I have more ownership of patients on house staff team.
2) Different structure by each attending in regard to notes, rounding, expectations.
3) Different expectations from different attendings. Some attendings don't make their instructions and expectations clear. For instance, an attending asks student, "Call department X for problem Y for patient 1" and then runs away c/o being busy, thinking those are clear directions. Please give students clear instructions. As in, "let's call Dept X for pt Z who presents with problem A." Any questions? Student will then ask, "Are we calling dept X and not Y because of patient's unstable vitals and down trending.
4) Short time spent during rounds.
Benefits:
1) I think this rotation allowed for the most learning I've had in M3 year!

**Figure 2:**
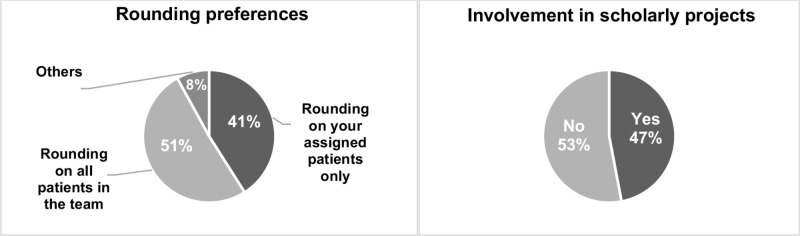
Perceptions of third-year medical students regarding the rounding preferences and their involvement in scholarly projects in a non-resident hospitalist rotation. ‘Others’ corresponds to the responses provided in the comment section.

Twenty students (41%) preferred rounding on only their own patients while a majority (25 students, 51%) preferred rounding on all patients in the team (Figure 2).

Three of the four comments pertaining to the rounding preference suggested a mixture of the two (Table 1). Almost half the respondents (23 students, 47%) stated that they had an opportunity to get involved in scholarly projects with their faculty hospitalist (Figure 2).

Table 2 shows the students’ perceived barriers to learning during a non-resident hospitalist rotation.

**Table 2:** Perceptions of third-year medical students regarding the barriers to learning in a non-resident hospitalist rotation. 'Others' corresponds to the responses provided in the comment section.

What are the barriers to learning in a hospitalist rotation?	
1) Variability in individual hospitalists’ rounding methods/times	42%
2) Lack of sufficient time for teaching due to clinical and administrative responsibility of the hospitalist	29%
3) Busy service	7%
4) No structured rotation	4%
5) Any of the above may act as a barrier	9%
6) Others	9%

**Figure 3:**
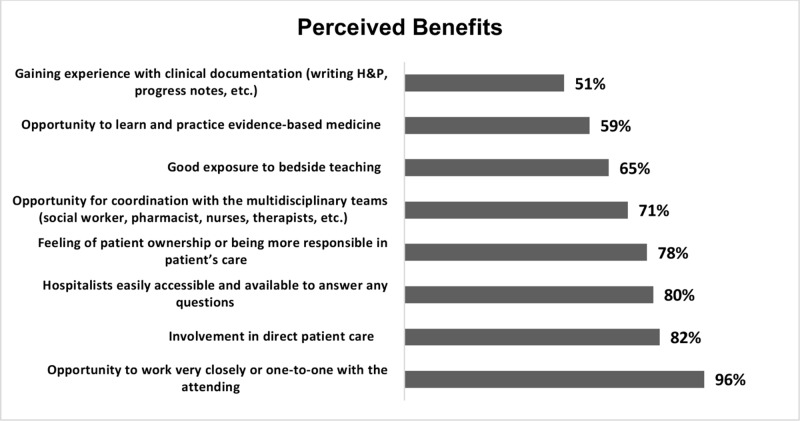
Perceptions of third-year medical students regarding the various benefits of a non-resident hospitalist rotation.

The major barriers to learning in the hospitalist rotation included the variability in individual hospitalists’ rounding methods/times, as reported by 19 out of 45 students (42%), followed by the lack of sufficient time for teaching as reported by 13/45 students (29%) (Figure 3). Four students were excluded from the analysis since they had missing responses. Other barriers included a busy service (7%) and “no structured rotation” (4%). Nine percent considered any of the aforementioned issues as possible barriers to learning. Further, nine percent provided free-written comments, as shown in Table 1.

Figure 3 demonstrates students’ perceptions regarding the benefits of rotating on a hospitalist service.

The students were asked to respond positively (“Yes”) or negatively (“No”) to each section separately. Opportunity to work very closely with the attending was regarded as the major benefit of rotating with a hospitalist (96%). Other perceived benefits included their involvement in direct patient care (82%), easy accessibility and availability of the hospitalist (80%), feeling of patient ownership (78%), opportunity for coordination with the multidisciplinary teams (71%), good exposure to bedside teaching (65%), opportunity to learn and practice evidence-based medicine (59%), and gaining experience with clinical documentation (51%).

## Discussion

Several conclusions can be made from the results of the study. Most commonly, students felt that a maximum of three patients should be assigned to them at a time during the rotation in a non-resident hospitalist team. This finding is remarkable, as there is a paucity of literature on the student perspective of the volume of patients and its impact on the educational value of the overall rotation. It is important to consider the students’ previous clinical experiences as a factor in their ideal patient volume. Students who rotate in internal medicine as their first clinical rotation may not be as comfortable with a large number of patient assignments as those who participate in internal medicine later on in their third year after they had accrued experience from other clerkships. This study did not account for the time at which students rotated through a hospitalist service relative to other rotations. Over the course of the rotation with a hospitalist, the student may become more comfortable with taking more patients as they learn the logistics of the rotation. Hence, we suggest that before assigning a particular number of patients to a student, hospitalists have to consider the specific student’s previous exposure to internal medicine rotations, individual comfort level, and the length of time the student has been on the rotation, with a general starting point of three patients on an average. Our findings reflect the need to adjust the volume of patients in order to maintain a fine balance between making the hospitalist rotation "educational" and preventing it from being "overwhelming" to the students. Our survey also showed that the majority of respondents (51%) favored rounding on all the patients in the team. This is apparently in contrast to the preference for fewer patients with 47% saying three patients would be ideal. This actually might reflect how much the students value teaching and learning during the rotation, as "carrying" and getting involved in the direct clinical care of fewer patients will allow more time for them to learn, albeit in fragments, from other patients in the team as well.

A majority of the students (57%) voiced their preference for taking care of patients with fewer comorbidities. This may give them a better chance to have a more complete picture of each patient and learn disease processes without confounding medical conditions. A contrasting argument could be made by the 29% who preferred patients who are more complex. Dealing with such complexity could provide more opportunity to learn more about a variety of disease processes and how they interact. The students who participated in the survey and decided to add comments seemed to be in agreement that a combination of both complex and simple patients was most beneficial for them. Students have very different learning styles, and some learning styles have even been shown to correlate with higher levels of success later in their education [12]. Due to this fact, we do not seek to suggest any strict guidance for the consideration of patient complexity on assigning patients to the students given that students constitute a heterogeneous group of learners. Further studies would be needed to correlate students caring for complex versus simpler patients and objective outcomes on student learning and performance and how this will differ between a resident versus a non-resident hospitalist team.

The students identified various barriers to learning while in the hospitalist team. The most frequently cited barrier (42% of students) was the variability between attendings’ rounding times. The unpredictability of rounding times reasonably can hinder the rounding timeliness and effectiveness, resulting in unnecessary consumption of time, which could have been effectively used for educational purposes otherwise. About 29% of students felt that the different clinical and administrative responsibilities of the hospitalist negatively impacted the time for their involvement in teaching students. This concern may be lessened by innovations in academic hospitalist work structure, including leveraging the census/workload on the hospitalist team and creating/re-structuring hospitalist workflow to balance clinical and teaching duties. Geographical localization is a hospital initiative to localize the patients of each hospitalist team to the designated hospital floor and this localization of patients can save time in rounding on the patients and facilitate more teaching time for the students. A study done by Olson et al. showed that the geographic localization of house staff patients improved patient-provider communication, satisfaction, and culture of safety [13], and there may also be a potential benefit of geographic localization on medical student education.

Only four percent of the students opined that the absence of structured rotation acted as a barrier to learning, though in the comment section, students expressed their concerns about not knowing how to call consults, perform other day-to-day tasks, and keep up with the different structures and expectations from each attending. It seems clear that defining expectations for students and assistance with day-to-day tasks is just as important to the students on non-resident hospitalist teams.

The perceived benefits of the non-resident hospitalist rotation were multifold. The single most important benefit was the opportunity to work closely or one-to-one with the attending, with 95% of students saying so. This is similar to our personal observations in interactions with the students. In comparison to the resident teams where the students likely spend more time with the residents, a non-resident hospitalist rotation provides a greater opportunity to work closely and directly with an attending. Other benefits that the students' value were: involvement in direct patient care, easy accessibility and availability of hospitalists, feeling of patient ownership, opportunity to get involved in coordination with the multidisciplinary teams, good exposure to bedside teaching, opportunity to practice evidence-based medicine, and gaining experience with clinical documentation. In addition, 47% of the students reported that they had the opportunity to do a scholarly project, including writing case reports during the rotation. The important role of scholarly work in medical education cannot be overstated. Under the direct supervision of the attending, the rotation can be a particularly good opportunity for the students to get involved in scholarly activities, which might include case-report writing on the interesting patients they are following or involvement in other clinical research projects. Hospitalists should encourage students to partake in scholarly activities, as they contribute to their overall educational experience and can also help in developing future physician-scientists [14].

One of the strengths of this survey was the optimal response rate. As with other online questionnaires, this survey was cost-effective with rapid data acquisition [15-16] Several limitations of this study should be considered. A well-documented limitation pertaining to most web-based surveys includes a potential decrease in validity and generalizability [15-16] Our survey was limited to a single institution, but larger comparative studies done in multiple institutions would be necessary for the results to be generalizable. Our study does not examine faculty perception of working with the medical students in hospitalist teams. The question regarding what hospitalists believe are the limitations in their teaching experience with medical students is pertinent to have a complete picture. Future studies reflecting the perspectives from the hospitalists will be needed.

Despite all the limitations, our study highlights an important, rarely discussed topic of the students’ perceptions regarding a non-resident hospitalist team and reflects the need for more structure in rotation and innovation in the curriculum, to enhance the goal of educating medical students.

## Conclusions

Since its inception in the 1990s, hospitalist medicine has grown to become an integral part of inpatient care today, and the academic hospitalist’s responsibilities extend beyond their clinical work, as they also play significant roles in medical education. With its continued expansion, it is important to better understand the unique roles of hospitalists as clinician educators. Our study examined the perceptions of third-year medical students regarding a non-resident hospitalist rotation and highlights many key issues that have the potential of making the rotation more educational for the students. Future large-scale studies are needed in this area to further define and optimize the educational experience of students on a hospitalist rotation.
